# RNA Sequencing Reveals the Inhibitory Effect of High Levels of Arachidonic Acid and Linoleic Acid on C2C12 Differentiation and Myogenic Biomarkers

**DOI:** 10.3390/nu16050706

**Published:** 2024-02-29

**Authors:** Wei Wang, Mohamed Abdelrahman, Ying Yang, Haimiao Lv, Liguo Yang

**Affiliations:** 1 Key Laboratory of Agricultural Animal Genetics, Breeding and Reproduction of Ministry of Education, Huazhong Agriculture University, Wuhan 430070, Chinahzyy666@webmail.hzau.edu.cn (Y.Y.);; 2 Animal Production Department, Faculty of Agriculture, Assuit University, Asyut 71515, Egypt

**Keywords:** fatty acids, muscle cells, myogenesis, omega-6, RNA sequencing, biomarkers

## Abstract

Over the past three decades, studies have shown that consuming polyunsaturated fatty acids (PUFAs) can enhance animal and human health and welfare through biological, biochemical, pathological, and pharmacological impacts. Furthermore, omega-6 plays key roles in the cardiopulmonary system, including promoting airway relaxation and inhibiting atherosclerosis and hypertension. However, findings from investigations of the effects of omega-6 fatty acids on molecular and cellular activity and discussions on their influence on biomarkers are still unclear. Therefore, the present study aimed to evaluate omega-6 fatty acids, the arachidonic acid (AA), and linoleic acid (LA) effects on C2C12 proliferation, myogenesis morphology, and relative myogenic biomarker expression through the Wnt pathway. C2C12 cells were cultured with and without 25, 50, 100, and 150 µM of LA and AA and then subjected to CCK8, Giemsa staining, RT qPCR, Western blotting, and RNA Sequencing. The CCK8 Assay results showed that 25, 50, 100, and 150 µM LA significantly decreased the viability after 72 h for 25, 50, 100, and 150 µM concentrations. Also, AA supplementation decreased cell viability after 24 h for 150 µM, 48 h for 150 µM, and 72 h for 50, 100, and 150 µM concentrations. Moreover, the LA and AA inhibitory effects noticed through Gimesa staining were morphological changes during myoblast differentiation. Both LA and AA showed inhibiting *IGF1*, *Cola1*, *Col6a2*, *Col6a1*, *Itga10*, *Itga11*, *SFRP2*, *DAAM2*, and *NKD2* effects; however, the depressing effect was higher for AA compared to *LA*. The previous results were confirmed through Western blotting, which showed that 50 µM LA and AA significantly reduced *DAAM2* and *SFRP2* protein levels compared to the control. Regarding RNA sequencing results, LA and AA increased the number of differentially expressed (DE) Mt-rRNA and snoRNA; however, the numbers of lncRNA detected decreased compared to the control. Our findings demonstrate that high and moderate LA and AA concentrations reduce primary myoblast proliferation and differentiation. Also, they highlight novel biomarkers and regulatory factors to improve our understanding of how the nutrition of fatty acids can control and modulate the myogenesis and differentiation process through different biomarker families.

## 1. Introduction

The omega-6 fatty acids are a group of polyunsaturated fatty acids (PUFA) distinguished by the presence of two or more cis-double bonds, with the first double bond six carbon atoms from the methyl end of the molecule, such as arachidonic acid (AA) and linoleic acid (LA) [1]. However, previous studies have demonstrated that consuming ω-6 can be dangerous if ingested in an unbalanced ratio with omega-3 fatty acids [2,3,4].

Omega-6 fatty acids are found naturally in Safflower, sunflower, and corn, rich in LA, besides soybean, sesame, and almonds. Although linoleic acid is present in all plant-origin oils but in different proportions, it is the dominant fatty acid in canola, peanut, and olive oils. Also, LA can be converted to different ω-6 fatty acids [5]. Dietary intake, ω-6 fatty acids, depending on the animal’s needs and metabolism, affect tissue composition health regarding the incidence of overweight and obesity, which should be considered when determining nutritional needs [6].

Animals use fatty acids as a source of energy and an essential component of their skeletal muscle cells, which is a significant location of fatty acid catabolism in animals [7]. Omega-6 fatty acids have two roles in the body: firstly, they are structural components of membranes that influence membrane function, and secondly, they are precursors of eicosanoids, which control renal and pulmonary function, vascular tone, and immune responses. Correspondingly, it was reported that supplements rich in LA during late gestation resulted in more significant average daily gain during the growing and finishing phases, greater body weights and hot carcass weights, more excellent marbling, and, consequently, a more significant percentage of choice carcasses [8]. These previous results highlighted the effect of omega-6 effect on animal health and growth.

Skeletal muscle comprises most energy expenditure, and sufficient muscle mass is essential for healthy aging [4,9]. Although the molecular, genetic, and systemic aspects of myogenesis are well understood [3,10], little is known about the role of nutrition signaling, particularly muscle cell activity. Nutritional factors are among the elements that influence skeletal muscle differentiation [11,12]. There is significant research on how the environment affects myogenesis [13,14], but few studies have examined how nutrition affects skeletal muscle differentiation [15]. Most of the research on nutrition and muscle development focuses on maintaining/preventing muscle mass loss or the maturation of differentiated muscle [16].

According to prior studies, the influence of various lipids and free fatty acids (FFAs) on skeletal muscle differentiation is still debatable, and the mechanism underlying this influence is still unclear, especially under obesity conditions [17].

The stages of life during which most developmental processes occur are the embryonic, fetal, and neonatal periods [18]. Animals deposit ω-6 PUFA through the direct intake, feeding, or body-based desaturation and elongation mechanisms that create longer-chain PUFA from short-chain FA precursors. Since the fetal stage is crucial for developing skeletal muscle and intramuscular adipocytes, omega-6 FA supplementation may alter myogenesis and, in turn, the sites where marbling will form later in life [19]. Therefore, the present study investigated the influence of omega-6 fatty acids on muscle cell development and their reflection on myogenesis biomarkers.

## 2. Materials and Methods

### 2.1. Cell Culture

C2C12 cells (from Procell, Wuhan, China) were cultured in growth medium (GM) containing Dulbecco’s Modified Eagle’s medium [+] 4.5 g/L D-Glucose, L-Glutamine [-] Pyruvate (DMEM, Hyclone, Logan, UT, USA), supplemented with 2% horse serum (Hyclone) and 1% penicillin–streptomycin (Biosharp, Beijing, China) at 37 °C under 5% CO_2_. The fatty acids (LA and AA) (YuanYe, Shanghai, China, Biotechnology Co. Ltd., Shanghai, China, Analytical standard, GC ≥ 95%) were dissolved in ethanol and combined with bovine serum albumin (BSA) in 0.2% BSA [20].

### 2.2. Cell Proliferation Assay

Cells were seeded in a 96-well plate at 2 × 10^3^ cells/well and cultured in a growth medium. The cells, 12 h after plating, were washed twice in PBS and treated with various concentrations (0, 25, 50, 100, and 150 µM) of LA or AA. The CCK-8 assay kit (CCK-8; Dojindo Laboratories, Kumamoto, Japan) quantified proliferating cells 24, 48, and 72 h after LA or AA treatment. Then, 10 µL of CCK-8 reagents were added to the cells for 1–4 h. Absorbance at 450 nm was measured using the multifunctional full-wavelength microplate reader (PE Enspire, PerkinElmer, Shelton, CT, USA).

### 2.3. Giemsa Staining and Differentiation Morphology

C2C12 cells were washed with PBS, fixed with 4% PFA for 10 min, and rinsed three times with fresh PBS for 5 min each time. The fixed cells were then incubated with Giemsa stain (Solarbio, Beijing, China) for 15 min. Cells were rinsed with PBS for 3 min and photographed using an inverted microscope.

### 2.4. qRT-PCR Measurement

According to the manufacturer’s instructions, total RNAs extracted from C2C12 cells were reverse-transcribed to cDNA according to the manufacturer’s instructions. SYBR Green Real-time PCR Master Mix reagents were used for real-time quantitative polymerase chain reaction (PCR), and PCR reactions were carried out on a CFX96™ Optical Reaction Module (CFX96, BIO-RAD, Hercules, CA, USA). The relative expression of mRNAs was normalized with GAPDH actin levels using the 2^−ΔΔCt^. The primer sequence was obtained from NCBI, as shown in Table 1.

### 2.5. Western Blotting

Protein concentration was determined using the BCA protein concentration assay kit (Biosharp, Beijing, China) after lysing cells in RIPA Buffer (Servicebio, Wuhan, China) and adding 100× phosphorylated protease inhibitor A solution, phosphorylated protease inhibitor B solution, 50× protease inhibitor PMSF, 50× protease inhibitor cocktail (Servicebio, Wuhan, China). The protein samples were electrophoresed in SDS Buffer (Omni-Easy One-Step PAGE Gel Rapid Preparation kit, EpiZyme, Shanghai, China), and then, the proteins were transferred to the PVDF membrane for the electrotransfer solution. The membranes were sealed with the sealing liquid for 2 h, and the primary antibody was incubated at 4 °C overnight. Finally, an ECL chemiluminescent substrate kit (Biosharp, Beijing, China) was used to develop the protein bands after the membranes had been treated with the secondary antibody for two hours. After that, it was placed on a Quick Chemi 5200, Monad, Suzhou, China, chemiluminescence detector to scan the band. β-actin protein reference normalization was used to analyze all the protein levels.

### 2.6. RNA-Sequencing and Data Analysis

C2C12 cells cultured in the growth medium were treated without (control) or with 50 µM LA and AA. Then, total RNA was isolated using the Trizol Reagent (Biosharp, Beijing, China), after which the concentration, quality, and integrity were determined using a NanoDrop spectrophotometer (Thermo Scientific, Waltham, MA, USA). Then, sequencing libraries were generated using the TruSeq RNA Sample Preparation Kit (Illumina, San Diego, CA, USA). The library fragments were purified using the AMPure XP system (Beckman Coulter, Beverly, CA, USA) to select cDNA fragments of the preferred 200 bp length, and DNA fragments with ligated adaptor molecules on both ends were selectively enriched using an Illumina PCR Primer mix in a 15-cycle PCR reaction. Then, products were purified (AMPure XP system) and quantified using the Agilent high-sensitivity DNA assay on a Bioanalyzer 2100 system (Agilent, Santa Clara, CA, USA). The sequencing library was then sequenced on a Hiseq platform (Illumina) by Shanghai Personal Biotechnology Co. Ltd. (Shanghai, China).

#### 2.6.1. Analysis Process and Transcriptome Analysis Flow

Initially, the platform sequences the samples and generates the original data (Raw Data) in FASTQ format. We utilized Cutadapt (v1.15) software to filter the sequencing data in order to obtain high-quality sequences (also known as “Clean Data”) for additional analysis. The sequencing data comprised several connectors and low-quality reads. Bowtie2 (2.2.6) generated the reference genome index, and the filtered reads were mapped to the reference genome with Tophat2 (2.0.14); the default mismatch was no more than 2. The distribution of mapped read alignment regions was computed.

Simultaneously, we used the R language Pheatmap (1.0.8) software program to perform bidirectional clustering analysis on all of the samples’ genes. With the Euclidean approach to calculating distance and the Complete Linkage method for clustering, we obtained a heatmap based on the expression level of the same gene in multiple models and the expression patterns of other genes in the same sample.

Next, we mapped all the genes to Terms in the Gene Ontology database and calculated the numbers of differentially enriched genes in each Term. The purpose of the GO enrichment analysis is to obtain GO functional terms with a significant enrichment of differentially expressed genes, thus revealing the possible functions of differentially expressed genes in the samples.

#### 2.6.2. Correlation between Samples

We used the Pearson correlation coefficient to express the correlation of gene expression levels between samples.

#### 2.6.3. PCA Analysis

We used the R language prompt function to perform PCA Principal Components Analysis on each piece based on the amount of expression. The volcano map of differentially expressed genes was drawn using the R language ggplots2 software package, Volcano map: the abscissa is log_2_FoldChange, and the ordinate is −log10 (*p*-value).

### 2.7. Statistical Analysis

For the cell proliferation assay, qPCR relative expression and Western blotting differences were tested using ANOVA and the unpaired *t*-test. The significance level was set at *p* < 0.05 for all data analyses. Then, for mRNA analysis, we utilized HTSeq (0.9.1) statistics to compare the Read Count values on each gene as the original gene expression, followed by FPKM to standardize the term. The DE Seq (1.30.0) was then used to assess the genes with differential expression under the following conditions: expression difference multiple (log2FoldChange > 1), significant *p*-value 0.05.

## 3. Results

### 3.1. The Effect of Omega-6 Fatty Acids (LA and AA) on Cell Proliferation

**Figure 1:**
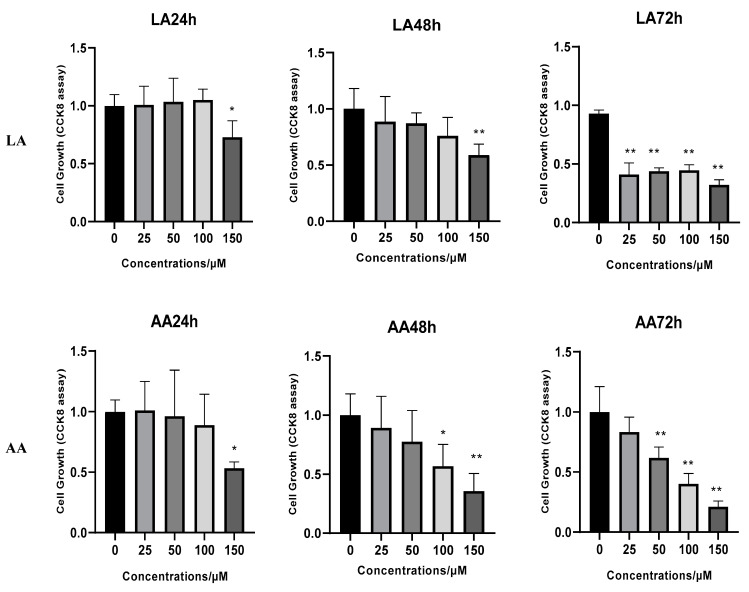
The effects of arachidonic acid (AA) and linoleic acid (LA) on the proliferation of C2C12 myoblasts. (*) indicates *p* < 0.05 treatment significant difference, (**) indicates highly significant.

As shown in Figure 1, according to the CCK8 Assay, the 25, 50, and 100 µM LA supplementation did not affect cell proliferation after 24 and 48 h; however, 150 µM significantly decreased the proliferation after 72 h for 25, 50, 100, and 150 µM concentrations. Also, AA supplementation decreased cell proliferation after 24 h for 150 µM, 48 h for 100 and 150 µM, and 72 h for 50, 100, and 150 µM concentrations.

### 3.2. The Effect of Omega-6 Fatty Acids (LA and AA) on Morphological Changes in C2C12 Myoblasts

**Figure 2:**
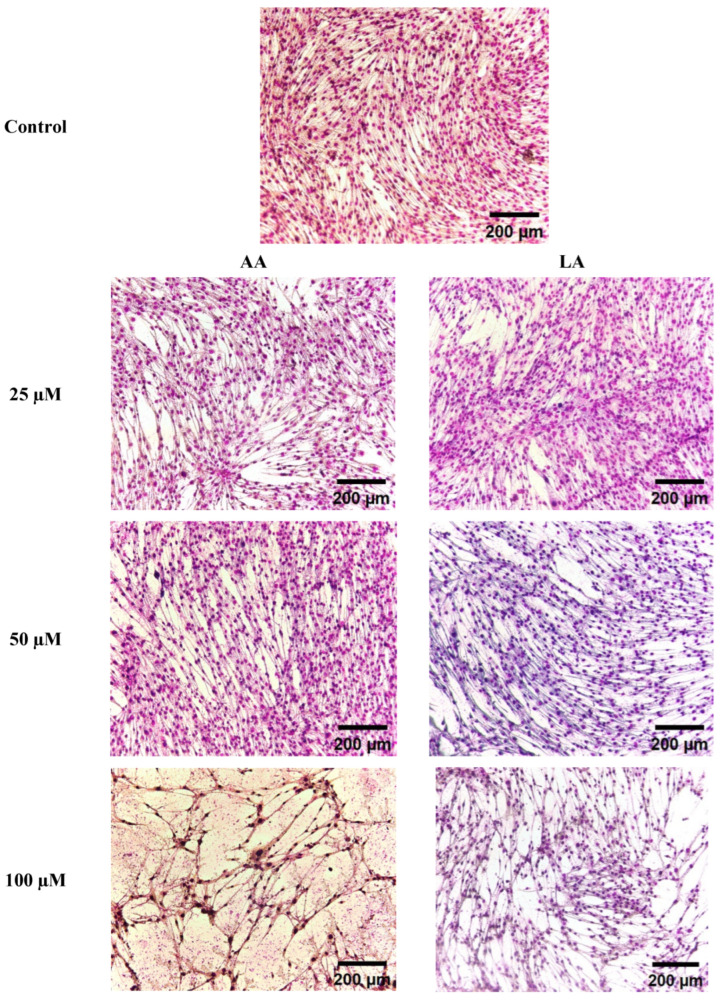

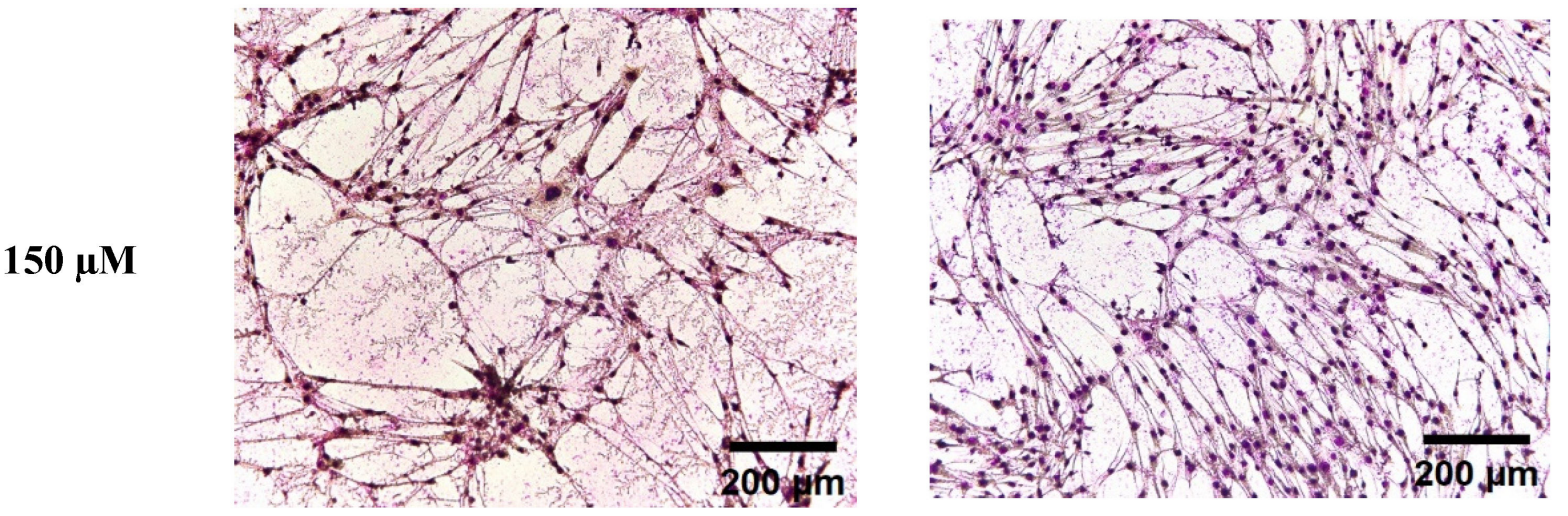
Giemsa staining presents C2C12 myoblasts after exposure to different LA and AA fatty acid levels during differentiation.

In this experiment, the LA and AA inhibitory effects noticed through Gimesa staining were morphological changes during myoblast differentiation. Figure 2 shows that the high levels of LA and AA reduced multinucleated myotubes, starting from 50, 100, and 150 µM, with high cell death in 100 µM and 150 µM. The changes can be seen in the decreasing nucleic number in myotubes; however, it shows that AA had higher depressing action than LA, as seen in Figure 2.

### 3.3. The Effect of Omega-6 Fatty Acids (LA and AA) on mRNA Expression of Myogenesis Biomarkers in the Wnt Pathway

**Figure 3:**
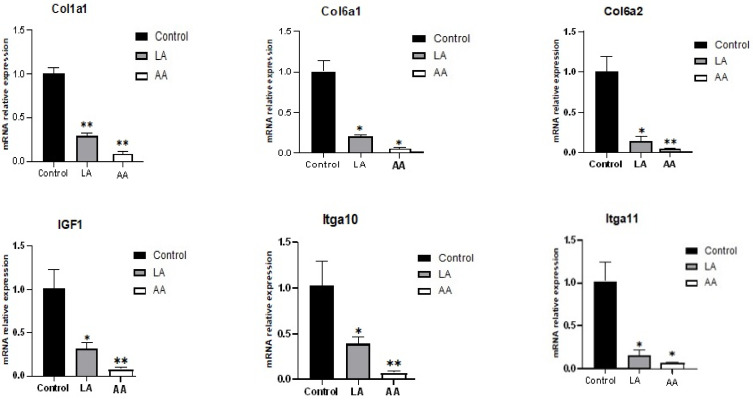

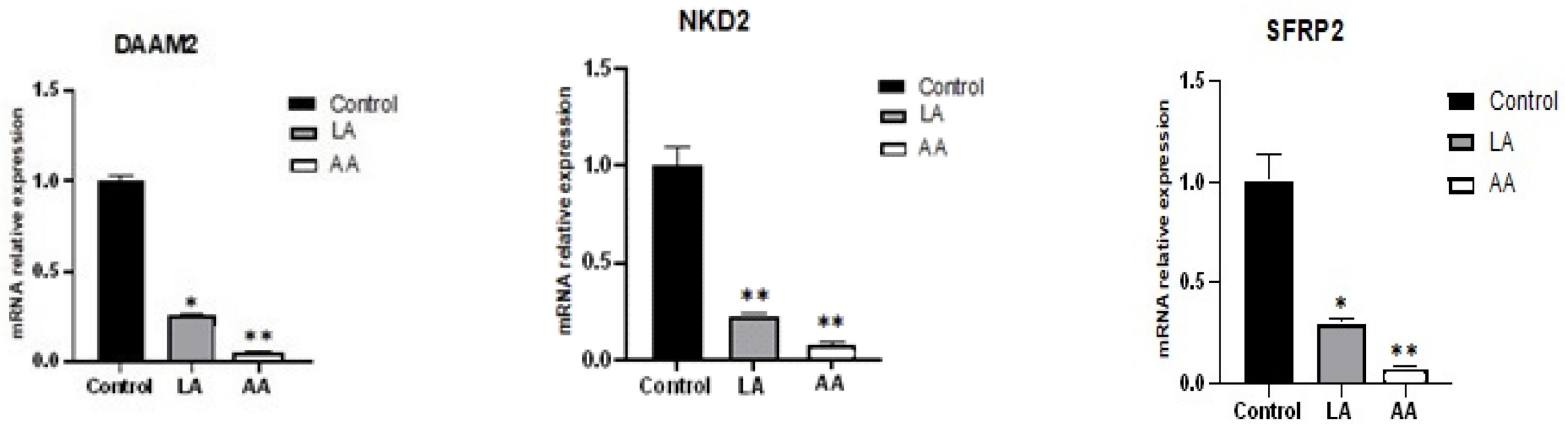
RT-qPCR gene expression for Wnt pathway myogenesis genes in C2C12 cells supplemented with 50 µM of LA and AA. (*) indicates *p* < 0.05 treatment significant difference, (**) indicates highly significant.

Figure 3 describes that the myogenesis genes were downregulated in C2C12 cells subjected to LA and AA treatments. The LA and AA groups exhibited a significant decrease in the *IGF1*, *Cola1*, *Col6a2*, *Col6a1*, *Itga10*, *Itga11*, *SFRP2*, *DAAM2*, and *NKD2* expression responding to 50 µM LA and AA. However, the depressing effect was higher for AA compared to *LA.* The *Col6a2*, *Itga10*, *SFRP2*, *IGF1*, *DAAM2*, and *NKD2* results showed that AA was highly significant in downregulating the mentioned genes.

**Figure 4:**
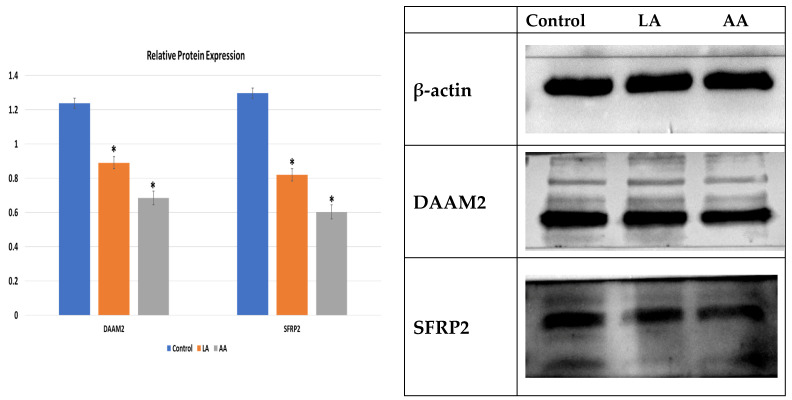
The relative protein expression for *DAAM2* and *SFRP2.* (*) indicates *p* < 0.05 treatment significant difference.

Also, as presented in Figure 4, immunoblotting was used to determine *DAAM2* and *SFRP2* at the translational level, showing that 50 µM LA and AA significantly reduced the *DAAM2* and *SFRP2* protein levels compared to the control. However, the results showed that AA had a higher depressing effect on myogenesis markers than LA.

### 3.4. RNA Sequencing Results

#### 3.4.1. Data Filtering

Table 2 presents the criteria for the data filtering process.

#### 3.4.2. Sample Correlation Test

**Figure 5:**
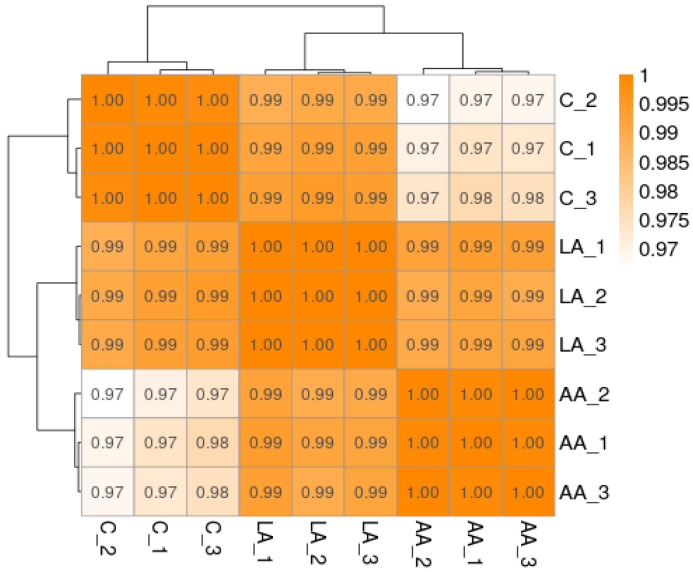
Pearson’s correlation coefficient representing the correlation of gene expression levels between samples.

Generally, a correlation coefficient between 0.8 and 1 is a strong correlation. If the correlation coefficient between samples of biological repeats is lower than 0.8, the correlation between the samples is low (Figure 5).

#### 3.4.3. PCA Analysis

**Figure 6:**
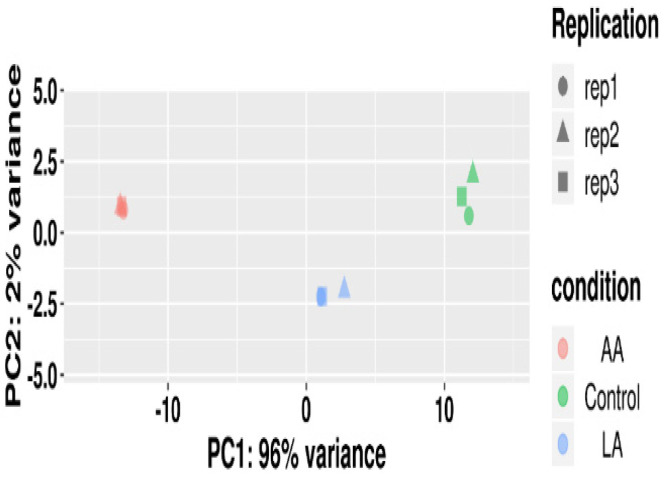
The corresponding relationship between replication and sample in the PCA diagram.

We used the DESeq2 software package of R language to perform PCA principal component analysis on each sample according to the expression level. The PCA analysis grouped similar samples; the closer the distance, the higher the similarity between samples, as shown in Figure 6.

#### 3.4.4. Analysis and Comparison of Differential Expression among Multiple Groups

**Figure 7:**
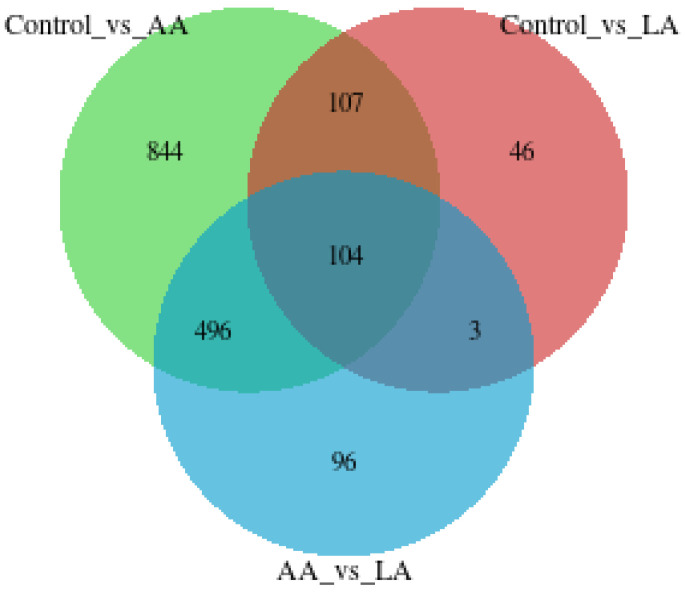
Venn diagram showing the number of different genes between the comparison groups and the overlapping relationship between the comparison groups.

**Figure 8:**
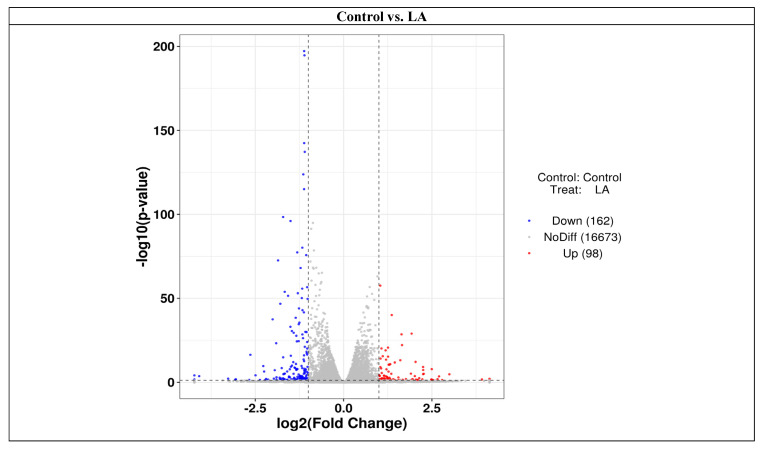

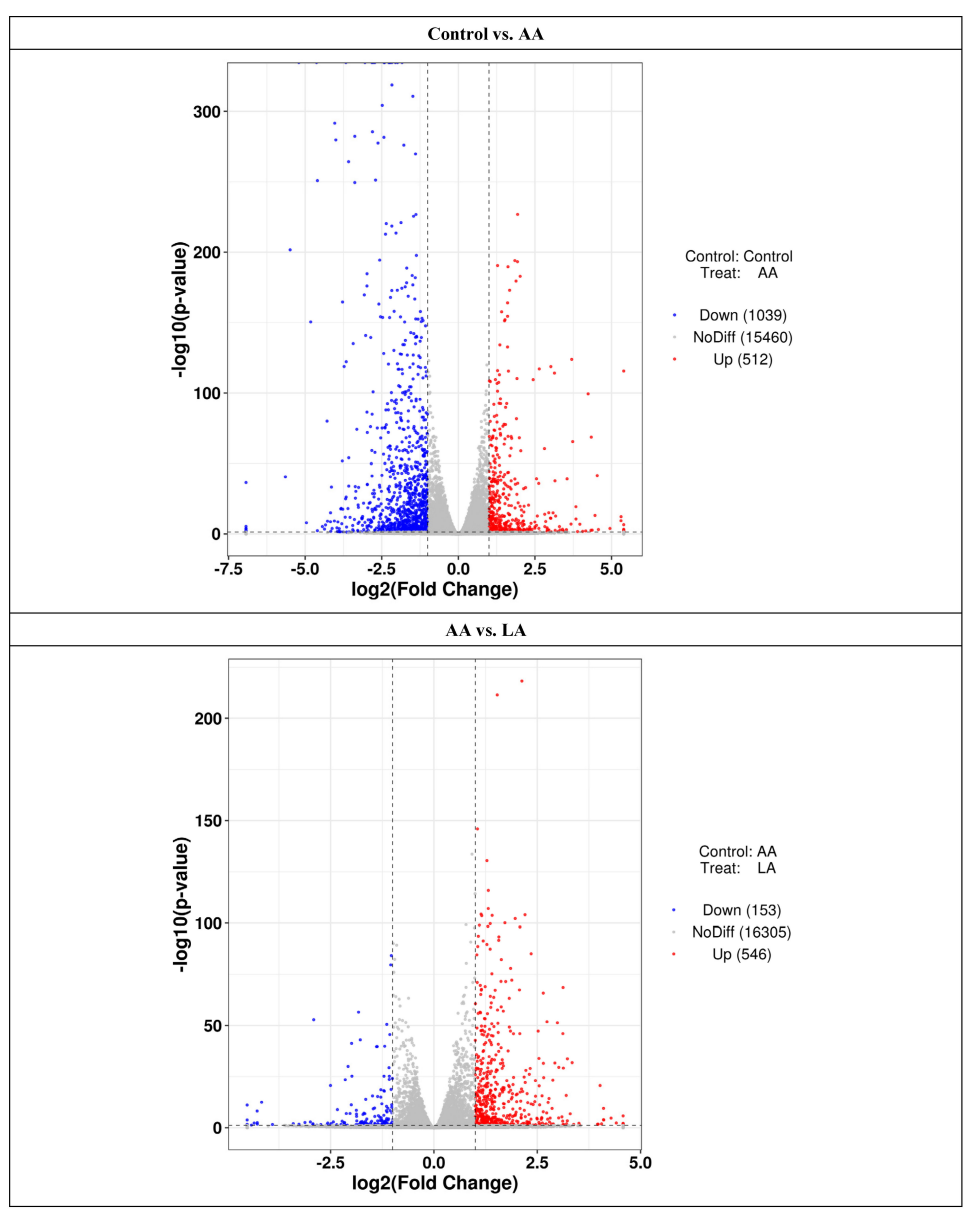
Volcano map for differentially expressed genes. The two vertical dotted lines in the figure are the 2-fold expression difference threshold; the horizontal dotted line is the *p*-value = 0.05 threshold.

As presented in Table 3 and Table 4, AA significantly triggered higher numbers of differentially expressed biomarkers than LA, including snoRNA, Mt_rRNA, and lncRNA. However, for omega-6 fatty acids, AA showed a higher effect on changing DEGs than LA, as shown in Figure 7 and Figure 8.

#### 3.4.5. Cluster Analysis

**Figure 9:**
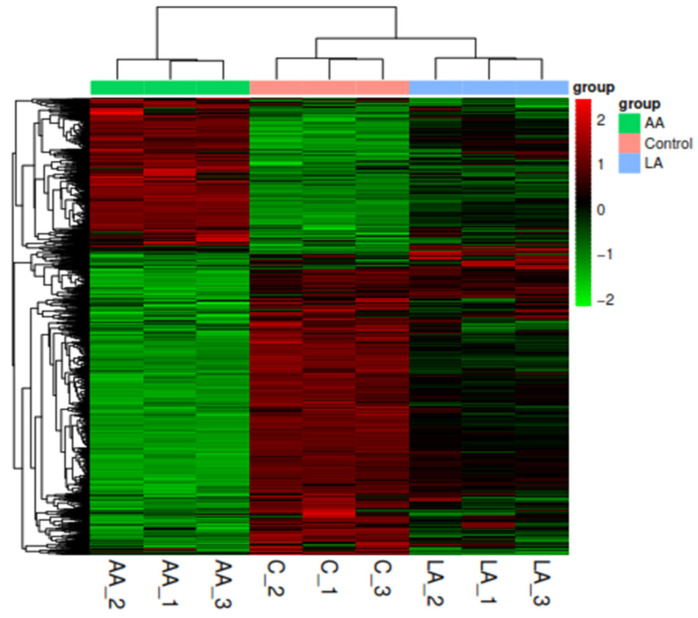
Cluster analysis evaluates the expression pattern of differentially expressed genes under different omega-6 fatty acids.

We can discover unknown biological relationships between genes involved in specific biological processes through expression clustering (Figure 9).

#### 3.4.6. Trend Analysis

**Figure 10:**
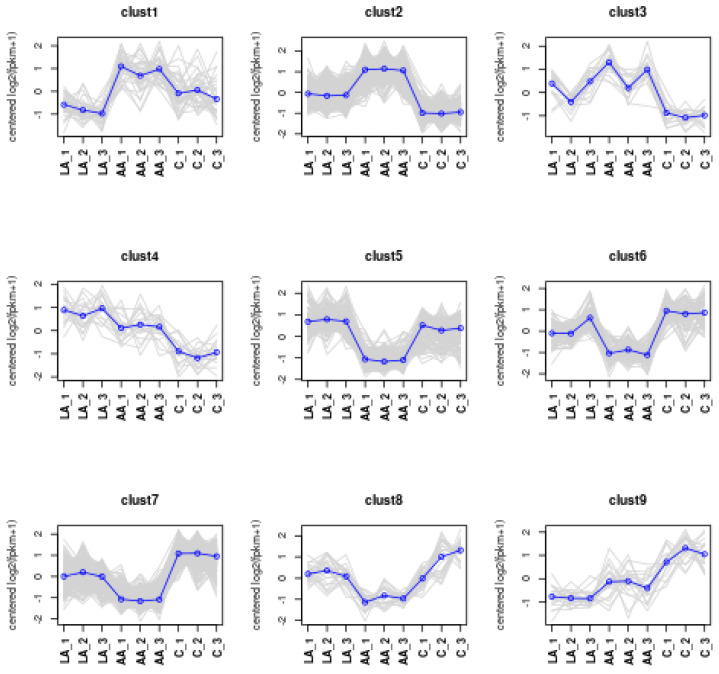
The expression pattern of genes in each cluster.

According to the cluster analysis results in the previous section, the differential genes according to the different expression patterns were used to obtain the clustering results of the genes (Figure 10).

#### 3.4.7. Function of Differentially Expressed Genes

1. Function analyzed through GO enrichment analysis

The enrichment of differential genes was compared with the whole-genome background to determine the main biological functions: molecular function (MF), physical process (BP), and cell component (CC).

**Figure 11:**
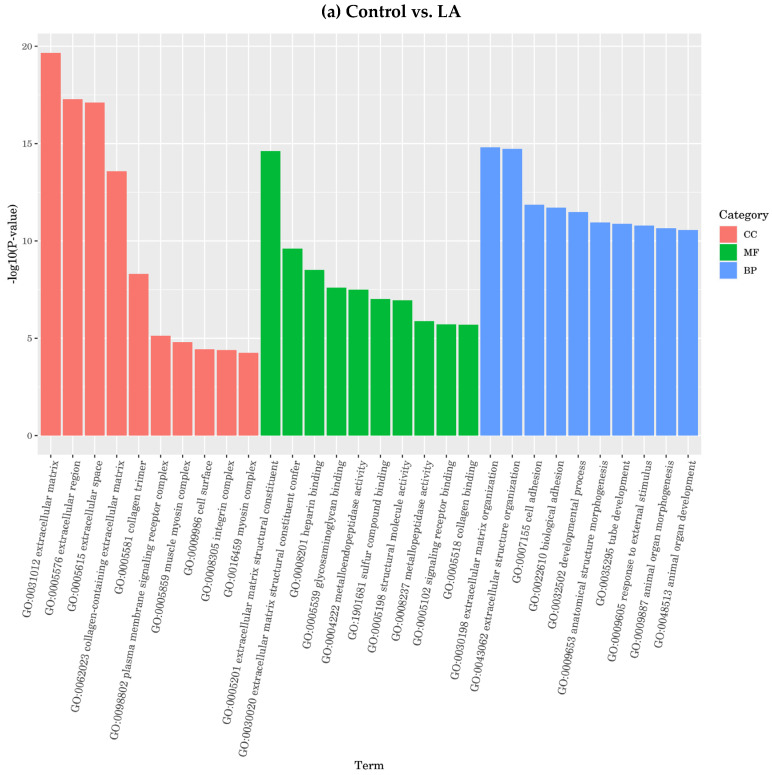

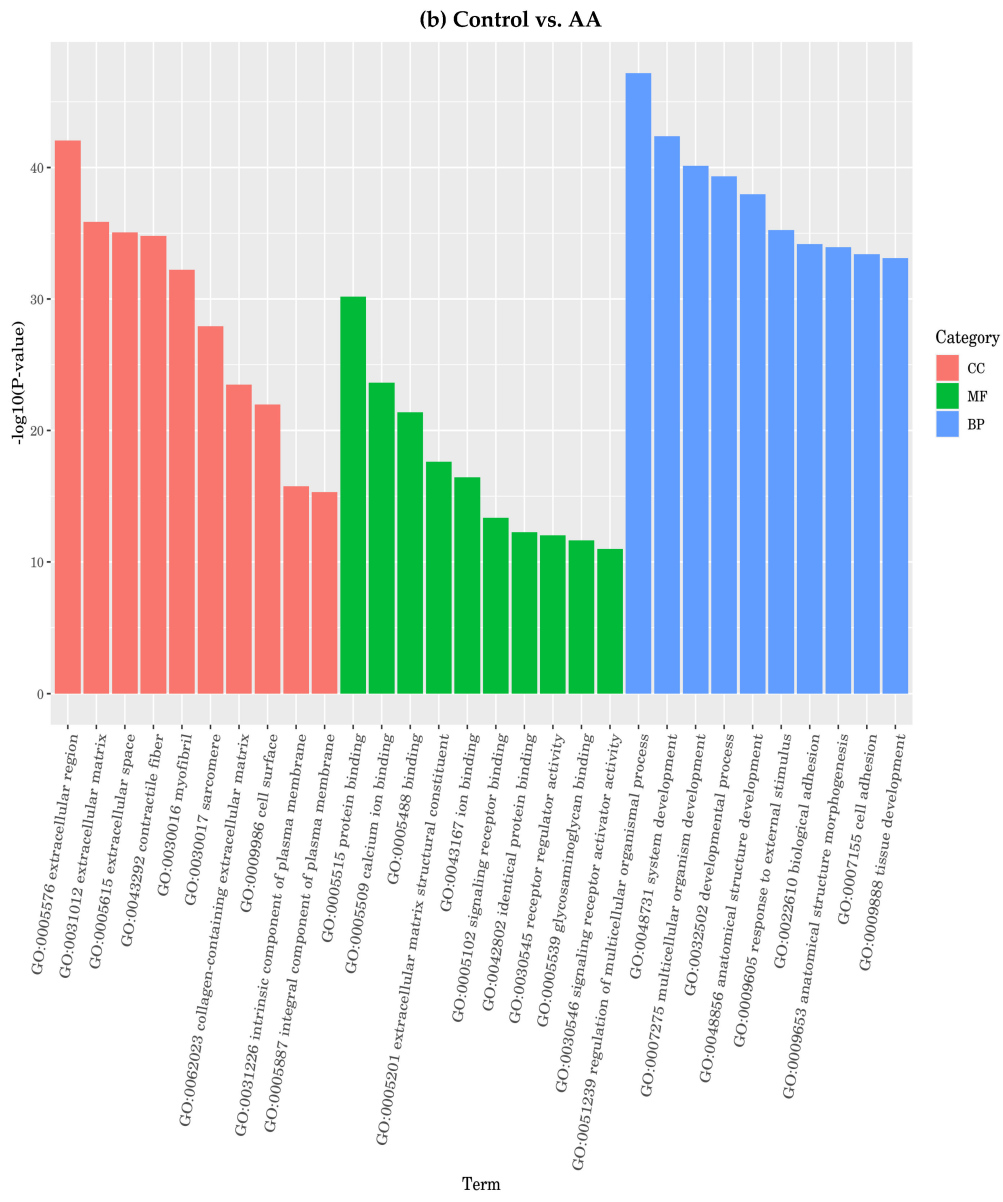

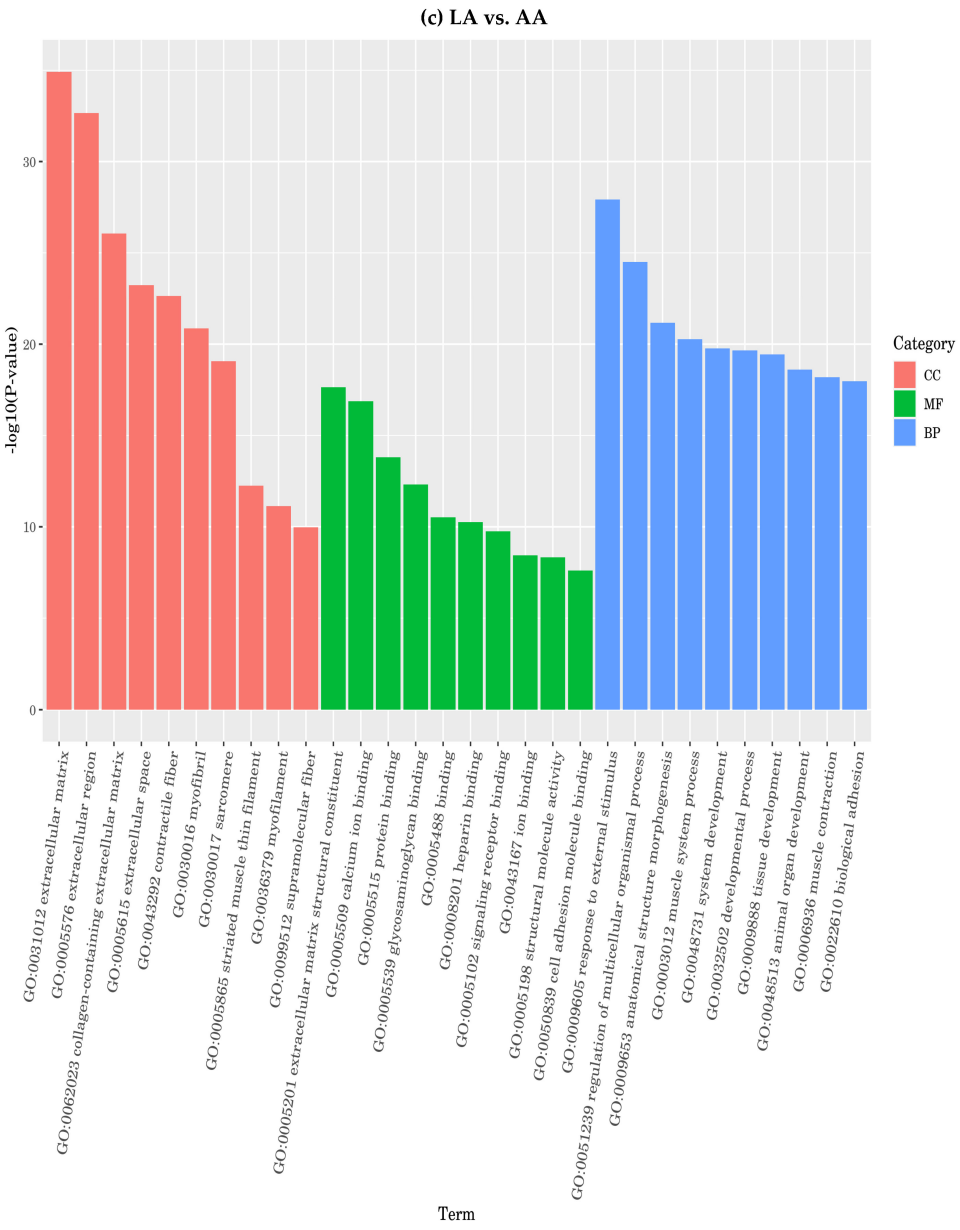
GO classification according to molecular function (MF), physical process (BP), and cell component (CC), selecting the most significant enrichment for omega-6 fatty acids.

On the other hand, omega-6 fatty acids LA and AA enriched different groups of functional genes. Compared to the control, LA enriched the cell component (CC) functions, including extracellular region, extracellular matrix, extracellular space, collagen-containing extracellular matrix, collagen trimmers, muscle myosin complex, integrin complex, myosin complex, cell surface, and plasma membrane signaling receptor complex, showing a higher potential of AA compared to LA (Figure 11). On the contrary, comparing LA to the control showed different molecular function (MF) genes, including extracellular matrix structural constituents, protein binding, signal receptor binding, calcium-ion binding, ion binding, receptor regulatory activity binding, identical protein binding, and signaling receptor activator activity. Also, regarding the physical process (BP) functions, LA enriched the extracellular matrix structural constituent, extracellular matrix structural constituent conferment, heparin-binding, structural molecule activity, signal receptor binding, metallopeptidase activity, glycosaminoglycan binding, metalloendopeptidase activity, and collagen binding. Also, regarding the physical process (BP) functions, LA enriched animal organ morphogenesis, extracellular matrix organization, extracellular structure organization, animal organ development, response to external stimulus, regulation response of stimulus, biological adhesion, development process, and cell adhesion.

Furthermore, AA compared to the control enriched cell component (CC) functions, including extracellular region, extracellular matrix, extracellular space, contractile fiber, myofibril collagen trimmers, muscle myosin complex, integrin complex, myosin complex, cell surface, intrinsic component for plasma membrane, and integral component for plasma membrane and sarcomere. Then, regarding molecular function (MF), AA enriched the extracellular matrix structural constituent, protein binding, signal receptor binding, calcium-ion binding, ion binding, receptor-regulator activity binding, and signaling receptor activator activity.

The physical process (BP) function AA enriched animal organ morphogenesis, extracellular matrix organization, extracellular structure organization, anatomical structure development, response to external stimulus, regulation response of stimulus, biological adhesion, regulation of the multicellular organismal process, system development, anatomical structure morphogenesis, and cell adhesion, as shown in Figure 11.

2. Gene Function analyzed through KEGG enrichment analysis

**Figure 12:**
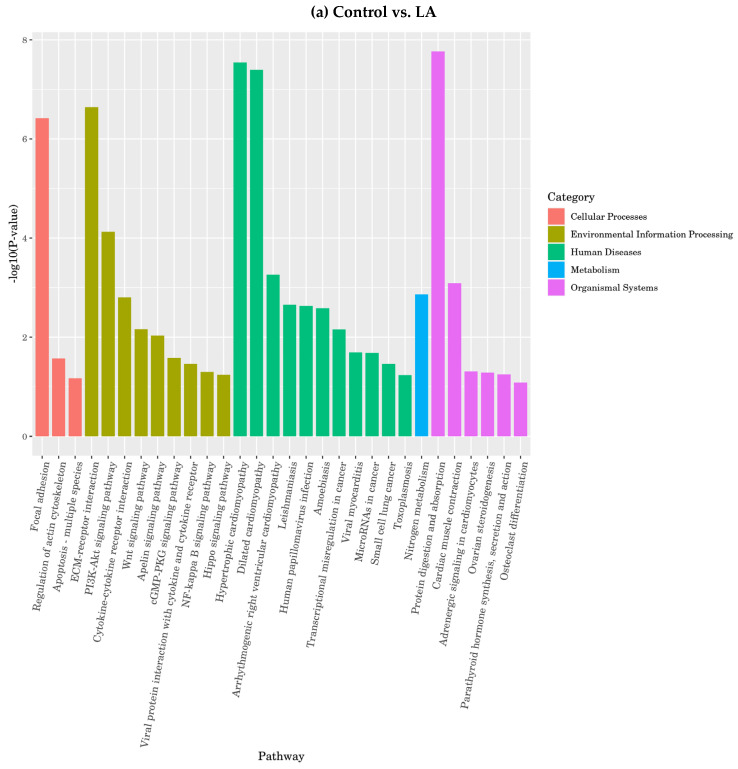

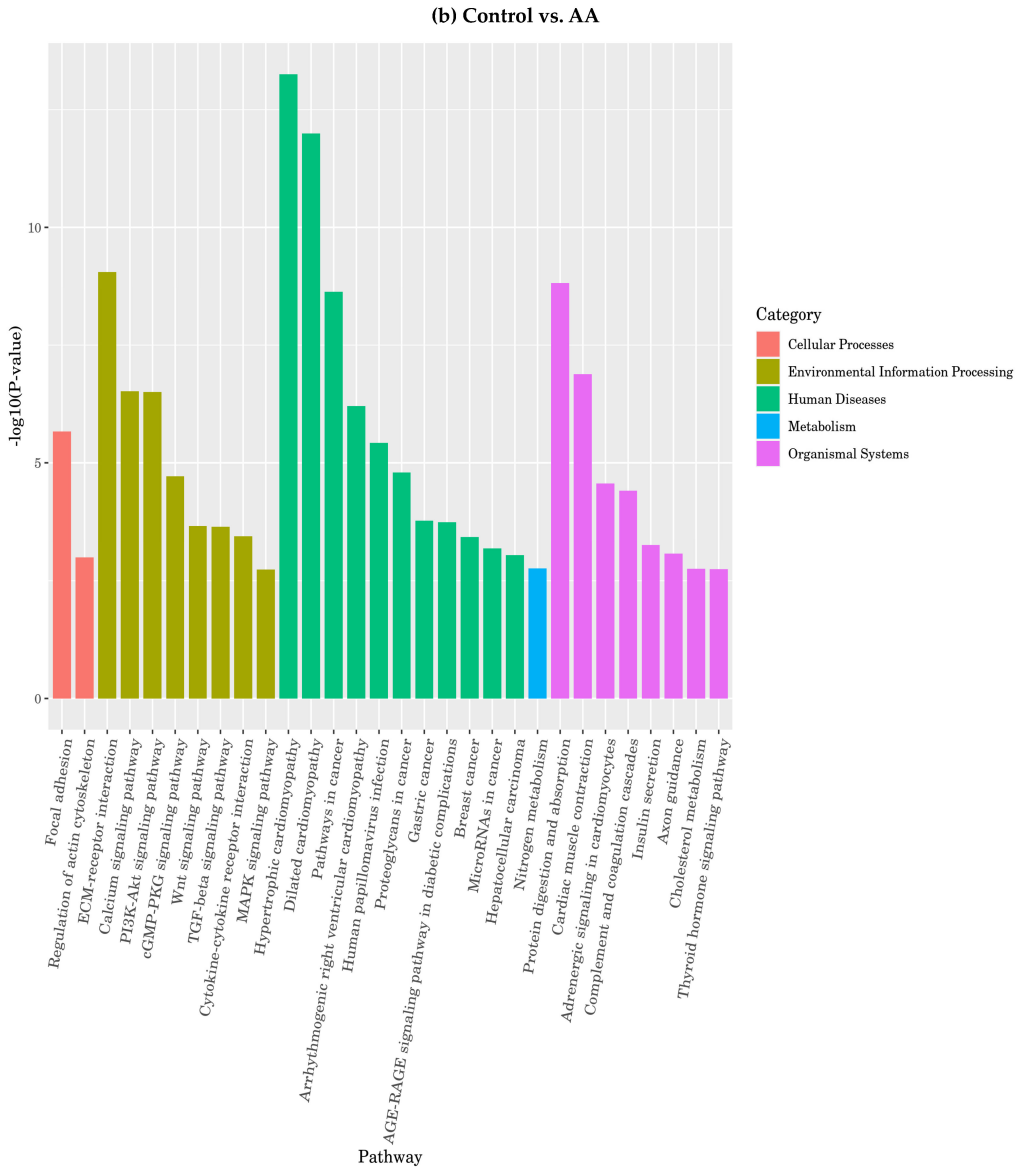

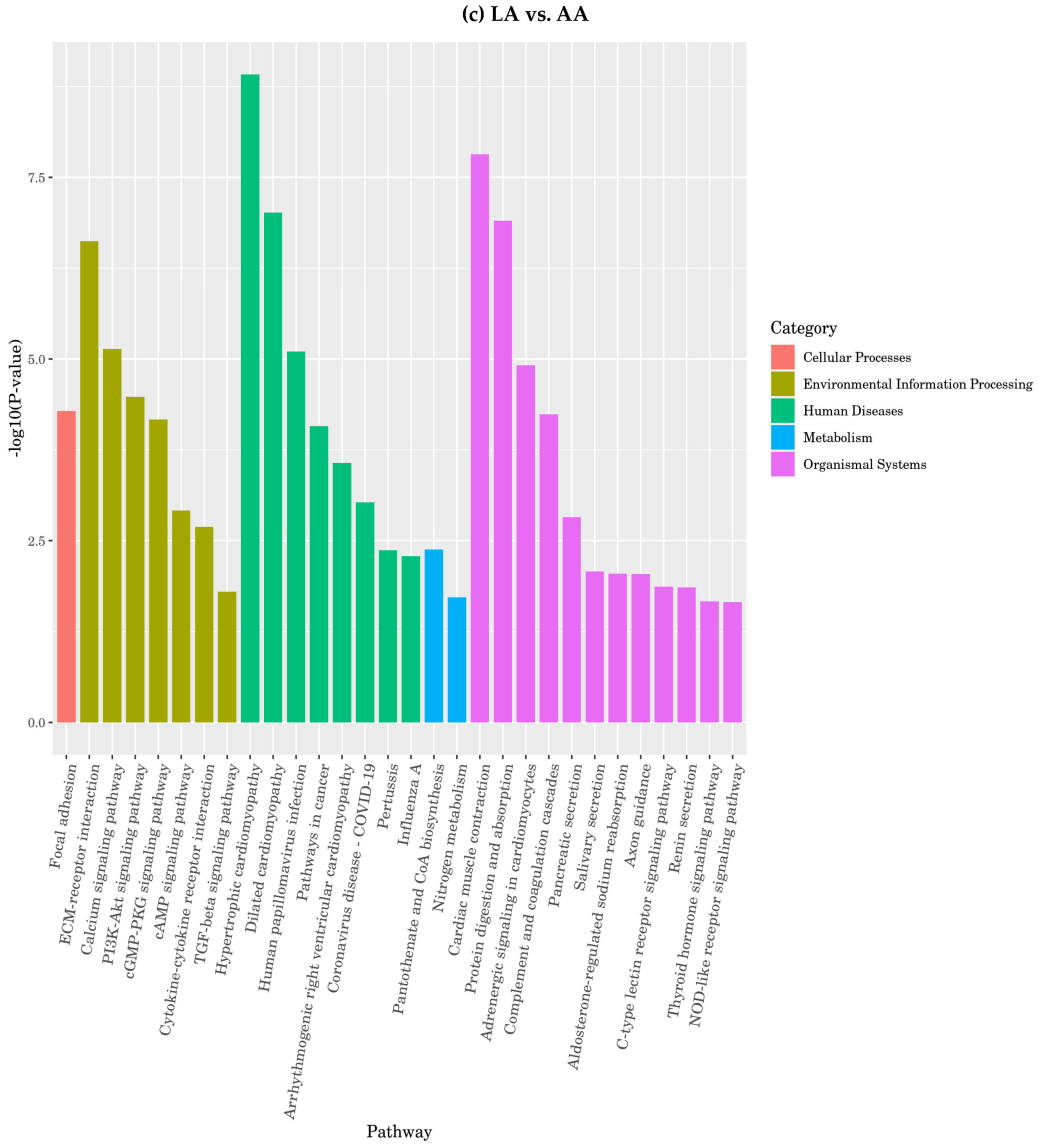
KEGG enrichment analysis for differentially expressed genes responding to omega-6 fatty acids.

The enrichment results for the omega-6 fatty acids showed that LA enriched focal adhesion, actin cytoskeleton regulation, and apoptosis in the KEGG pathway. Meanwhile, the most enriched pathways among environmental information processing functions were ECM-receptor interaction, cytokine–cytokine receptor interaction, and the WNT signaling pathway. Regarding metabolism, nitrogen metabolism was the most enriched function in the pathway. However, protein digestion and absorption, cardiac muscle contraction, adrenergic in cardiomyocytes, and insulin secretion were the most enriched functions in the organismal systems’ gene family, as presented in Figure 12.

## 4. Discussion

Myoblasts undergo a divided fusion response due to the actions of myo-marker and myo-regulators at various membrane remodeling stages. Cell fusion is crucial for the growth of multicellular animals and is a significant factor in developing different cell types and tissues. Recent research has brought attention to the diverse protein machinery characterized by various structural and functional components that cause plasma–membrane mergers in many systems. We focused on identifying and developing several significant sets of fusion proteins that collectively present a changing view of cell membrane fusion [21]. We also highlighted recent findings on developing vertebrate myoblast fusion in skeletal muscle, which comprises multiple multinucleated myofibers created by fusion progenitor cells.

Type I collagen is the major component of muscle cells, providing tensile strength. To accomplish the varying quantities of type I collagen in distinct muscle tissues throughout development, growth, ageing, and tissue healing, the profibrotic genes type I collagen (*Col1a1 and Col1a2*) are thought to be under complex transcriptional and posttranscriptional regulations. The *Col1a1* gene contains positively and negatively active genomic elements in tissue-specific transcriptional regulation [22]. Furthermore, the COL6 protein has been shown to interact with integrins, cytokines, and growth factors, and it is thought to be involved in cell proliferation, differentiation, and regeneration. Its primary function is to link the basement membrane to fibrous connective tissue.

Interestingly, by the present study’s results, it is the first study that found that LA and AA downregulated the expression of *Itga10*, *Itga11*, *Col1a1*, *Col6a1*, and *Col6a2*, which may be involved in muscle cell division and proliferation, and it also found that their control on DNA methylation can affect how differently muscles evolve [23]. The later downregulated genes were suggested as candidate genes for functions related to muscle development in farm animals, which are related to an increased number of muscle fibers and the regulation of late myogenesis [24]. Furthermore, *IGFs* control and promote protein synthesis, cell differentiation, hypertrophy, and cell proliferation during myogenesis. *IGF-I* and *IGF-II* were increased during differentiation in porcine satellite cells [25]. *IGF-II* mRNA was most elevated in fetal sheep on gestational day 85, highlighting its significance during this time of differentiation and the development of myogenic fibers in the leg [26], besides inducing muscle cell hypertrophy by increasing myotube diameters [27].

In the present study, LA and AA supplementation depressed *IGF1* expression, which is supported by previous findings that reported that LA stimulated differentiation at low concentrations (up to 50 μM) but inhibited differentiation at high concentrations (200 μM) [28].

Several growth factors, including insulin-like growth factor-1 (*IGF-1*), *IGF-2*, and IGF-binding proteins (*IGFBPs*), are crucial for excessive fetal growth [29]. *IGF-1* is one of these growth hormones that significantly contributes to excessive fetal growth. It can act as a paracrine factor by being released into the fetal circulation and as a paracrine factor by promoting fetoplacental development [29]. Fetal mice lacking the *Igf-1* gene weighed just 60% of their wild-type counterparts [30] because the placenta begins producing and secreting *IGF-1* early in pregnancy [29]. According to a study on Wistar rats, *IGF-1* and *IGF-2* are primarily expressed in the placenta; however, a study on ovines suggested that *IGF-1* may also be expressed in the fetal liver and skeletal muscles in late pregnancy. These results explain that LA and AA used concentration-inhibited myogenesis through integrative depression for cytokines and collagen regulation factors (*COL*), decreasing the cell’s profibrotic potential and inhibiting muscle fibers formation. These findings are supported by previous findings by Roberts et al., who reported that low concentrations of AA, especially those under 25 µM, led to myotube hypertrophy. In comparison, high concentrations (50–100 µM) caused myoblast death [31], while Takenaka-Ninagawa et al. described *COL6*’s role in changing muscle development and maturation [32].

*Sfrp2*, which is among the five proteins that make up the secreted frizzled-related protein (*SFRP*) (*Sfrp* 1–5), has striking similarities to the trans-membrane frizzled (Fz) receptors and can bind extracellularly to Wnts and inhibit Wnt signaling by attaching to the Fz receptor. *SFRPs* can prevent Wnts from attacking by competing with them. It is known that fibroblasts expressing high levels of *SFRP2* represent a significant population of muscle fibroblasts, which regulate *NKD2* expression [33]. Simultaneously, Guo et al. identified *SFRP2* as a candidate gene linked to pig growth features using RNA sequencing and existing livestock genomics research [34]. In mice, *SFRP2* has been found to play essential roles in muscle development, muscle satellite cell proliferation, and muscle satellite cell differentiation [35,36]. In pigs, *SFRP2* has been demonstrated to influence skeletal muscle formation during embryogenesis [34].

Also, the results showed that LA and AA depressed *DAAM2* protein, the member of the mammalian diaphanous-related formins, a disheveled-associated activator of morphogenesis 1 (*DAAM1*), which is ubiquitously expressed [37]. In addition to mediating the non-canonical Wnt/PCP (planar cell polarity) signaling pathway, the *DAAM* family has been discovered as a disheveled interaction factor [38]. *Daam* may promote actin remodeling during vertebrate gastrulation, neuronal dendrite stability, the development of filopodia, oocyte meiosis, and other processes that may improve cell motility [39]. Our earlier research revealed that active *DAAM* is necessary for Wnt5a-induced cell migration [40]. The therapeutic target *DAAM* may effectively promote cytoskeleton rearrangement and cell motility; however, no metabolite or antibodies can neutralize it [40]. Also, *DAAM1* and *2* are co-required for sarcomere assembly and myocardial maturation and are autonomously required for cardiomyocyte polarity and adhesion [41], and it was suggested that an early response to *DAAM1* stimuli partially induces fibroblast-mediated contractile activity [42]. The present study provides the first evidence of the potential inhibitory actions of high omega-6 fatty acids (LA and AA) on *DAAM2* and highlights their role in the myogenesis cycle, myoblast differentiation, and proliferation.

It has been proposed that consuming high dietary AA levels may induce inflammation since AA is the precursor to prostaglandins and leukotrienes, which are crucial components of the local inflammatory response [43,44]. It has recently been proposed that excess dietary ω-6 PUFA can exacerbate inflammation by causing the linoleic acid to produce specific oxylipin metabolite. Furthermore, linoleic acid may stimulate arachidonic acid pathways, increasing pro-inflammatory arachidonic metabolites produced by lipoxygenase (LOX) (such as leukotriene-B4) and cyclooxygenase (COX) (such as prostaglandin-E2 and thromboxane-B2). Oxidized linoleic acid activates Nuclear Factor–Kappa Beta (NF-kB), a transcription factor that causes inflammation. Thus, oxidized linoleic acid may cause inflammation in healthy intestinal, smooth muscle cells. Thus, the omega-6 polyunsaturated fatty acid linoleic acid may have proinflammatory properties, especially in those with inflammatory bowel disease [45].

However, according to other research works, a higher overall ω-6 PUFA intake has been linked to reduced or no inflammation, an improved blood cholesterol profile, and a lower risk of coronary heart disease. According to a previous study, the concentration of omega-6 myoblasts affects the degree of skeletal muscle differentiation. The study also found that although palmitic acid had little impact, oleic acid encouraged differentiation; while linolenic acid had little impact, docosahexaenoic and eicosapentaenoic acids prevented the proliferation of C2C12 cells [46].

In prior work, Markworth et al. discovered that the treatment of omega-6 fatty acids affected C2C12 skeletal muscle cells in a dose-dependent way [47]. The authors mentioned that low concentrations of AA, especially those under 25 µM, led to myotube hypertrophy, while high concentrations (50–100 µM) caused myoblast death. According to the findings of these studies, modest levels of omega-6 may have beneficial effects on skeletal muscle growth, whereas excessive levels may be harmful. Our results are supported by Son et al. who demonstrated that LA dramatically increases the expression of ANGPTL4 at both the gene and protein levels during skeletal muscle development. ANGPTL4 is also essential for LA to diminish myotube formation and downregulate Wnt/β-catenin signaling. These findings indicate that LA-induced ANGPTL4 impairs skeletal muscle differentiation. However, our findings show that AA harms skeletal muscle at supraphysiological levels more than LA. The contradictions in the results may be attributed to the difference in molecular mechanisms between smooth and skeletal muscles. Therefore, the results of previous studies suggest that additional research is needed to determine whether varied AA doses and data-collecting timing influence the different muscle cell types. It may also be that the low doses are more preferred and recommended for omega-6 fatty acids studies compared to omega-3, which can be studied in higher levels, and muscle cells tolerate higher levels of omega-3 compared to omega-6 and may tolerate one omega-6 fatty acid more than another one.

Our study has some limitations: we did not specifically explore all the potential mechanisms by which omega-6 fatty acids could influence proliferation and myotube fusion, which will be a focus of future work. On the other hand, a strength of our study is the data showing the transcriptional and protein data on *DAAM2 AND SFRP2*, indicating that only high AA doses decreased this protein; these data and our recent findings indicate that *DAAM2* and *SFRP2* are critical to skeletal muscle cell physiology. The specific biochemical roles of tissue polarity genes in the developing vertebrate’s central muscle system, and the complex network of interactions between these genes are still poorly known, although the number of these genes is increasing. This is the first study examining how high levels of ω-6 PUFA supplementation affect muscle tissue. Higher AA supplementation doses or extended AA supplementation periods may increase the amount of AA incorporated into muscle phospholipids. However, it is crucial to note that consuming various PUFA at the same absolute levels can have very diverse effects.

Our results show that high and moderate concentrations of LA and AA decrease myoblast differentiation and primary myoblast proliferation. Alterations to the Wnt pathway’s signaling may partially cause decreased proliferation, although other pathways may control AA-mediated decreases in differentiation. High AA levels may influence pathways mediating cell survival and death, favoring cell death processes, including apoptosis or necrosis. Such responses may be caused by the toxicity of AA and LA itself or by inhibiting essential survival signaling pathways.

Correspondingly, from the RNA sequencing results, high levels of LA and AA induced significant changes in both mitochondrial rRNA (Mt-rRNA) and long noncoding RNA (lncRNA). According to previous evidence, long non-coding RNAs (lncRNAs), not translated into proteins, have emerged as novel regulators of various biological processes over the past ten years [48]. In mitochondria, the organelles in charge of converting energy and producing adenosine triphosphate in eukaryotic cells, mitochondrial ribosomes (mitoribosomes), carry out protein synthesis. However, their role in cellular metabolism regulation during different cellular processes, such as myogenesis development, needs deep investigation and can be considered a novel research area as a candidate biomarker in molecular metabolism studies. As the current results showed, PUFA treatment for omega-6 fatty acids resulted in significant changes in small nucleolar RNA (snoRNA) signatures that can be promising points for novel future studies regarding and enhancing our understanding of nutritional regulation for myogenesis. snoRNAs are extensively found in the nucleoli of eukaryotic cells and range in length from 60 to 300 nucleotides, which is mainly connected to the alteration of RNAs, such as the AC4C of 18 SrRNA and the 2′-O-methylation and the pseudouridylation of rRNAs, and which can also control alternative splicing similar to miRNAs [49]. However, it is still unclear how snoRNA molecules affect muscle cell response to PUFA and how they modulate myogenesis and differentiation.

Finally, although different from earlier findings in murine C2C12 cells, primary myoblasts’ responses to AA and LA supplementation dosages are consistent with prior studies [50].

## 5. Conclusions

The present findings are the first to demonstrate that LA and AA can influence how differently muscles evolve by reducing the expression of genes such as *Itga10*, *Itga11*, *Col1a1*, *Col6a1*, and *Col62*, which may be involved in muscle cell division and proliferation. A more significant number of muscle fibers and the control of late myogenesis were two functions associated with the later downregulated genes’ potential roles in farm animals’ muscle growth. Omega-6 (LA and AA) supplements were discovered to reduce *IGF1* expression and control and promote protein synthesis, cell differentiation, hypertrophy, and cell proliferation during myogenesis. Our findings demonstrate that high and moderate LA and AA concentrations reduce primary myoblast proliferation and differentiation. While additional pathways may control AA-mediated declines in differentiation, modifications to the Wnt pathway’s signaling may partially result in a lower proliferation. Also, it highlights novel biomarkers and regulatory factors to enhance our understanding of how the nutrition of fatty acids can control and modulate the myogenesis and differentiation process through different biomarker families. However, each biomarker must be investigated separately to gain a deeper understanding and rely on much clearer evidence.

## Figures and Tables

**Table 1:** Primer sequences used for real-time qPCR.

Gene Name	Primer Sequences	Accession Number	Product Size (bp)
Itga11	F 5′ CAGCCTTTGGCCAGGATTCA3′	NM_176922.6	159
	R 5′ CCATTGGTTTCCATTGGGGC3′		
Col6a2	F 5′CCCTAACAGGAACCTAAACGAA3′	NM_001347207.1	100
	R 5′ GGTAGAGTCGGGTCGCATG3′		
Col6a1	F 5′ACACTCAACGGGACACGAC3′	NM_009933.4	147
	R 5′GCCGACCTTGCGATAAGC3′		
IGF1	F 5′GGACCGAGGGGCTTTTAC3′	NM_001111274.1	163
	R 5′TAGAGCGGGCTGCTTTTG3′		
Col1a1	F 5′AGAGCCTGAGTCAGCAGATTG3′	NM_007742.4	138
	R 5′AGCCTTGGTTAGGGTCGA3′		
Itga10	F 5′ATCTCTGGCAATGCAAGCTG3′	NM_001302471.1	242
	R 5′AAGGTGCTGACCACTGTCAC3′		
NKD2	F 5′ACAACCGCCAAGAATGGACAT3′	NM_001347535.1	99
	R 5′CCTCGTAGATGGTGTGCATCA3′		
SFRP2	F 5′CCCCTGTCTGTCTCGACGA3′	NM_009144.2	131
	R 5′GTCGCACTCCAGCATGTCT3′		
DAAM2	F 5′TGACCTTCCCGAGATCGACC3′	NM_001008231.2	103
	R 5′CTCTGCAAAGCGGACATTGAG3′		
GAPDH	F 5′AGGTCGGTGTGAACGGATTTG3′	NM_001289726.2	123
	R 5′TGTAGACCATGTAGTTGAGGTCA3′		

**Table 2:** Sequencing data filtering statistics.

Sample	Clean Reads No.	Clean Data (bp)	Clean Reads%	Clean Data %
C_1	44,560,604	6,728,651,204	94.61	94.61
C_2	41,755,710	6,305,112,210	94.69	94.69
C_3	48,940,596	7,390,029,996	94.63	94.63
LA_1	42,811,766	6,464,576,666	94.62	94.62
LA_2	45,734,926	6,905,973,826	94.64	94.64
LA_3	40,923,626	6,179,467,526	94.58	94.58
AA_1	44,172,092	6,669,985,892	94.56	94.56
AA_2	40,074,740	6,051,285,740	94.59	94.59
AA_3	48,299,736	7,293,260,136	94.58	94.58

**Table 3:** The expression difference between two groups treated with LA and AA.

Comparison	Upregulated	Downregulated	Total
Control vs. LA	98	162	260
Control vs. AA	512	1039	1551
AA vs. LA	546	153	699

Control: Control group samples; Treat: Experimental group samples; Upregulated genes: Treat upregulated genes compared to control; Downregulated genes: Treat downregulated genes compared to control; Total DEGs: Treat compared to control differentially expressed genes.

**Table 4:** The statistical analysis for different gene biomarkers (expressed as means ± SD); differences were significant when (*p*-value < 0.05).

Biomarker	Control	LA	AA	*p-*Value
Mt_rRNA	102,59.67 ^a^ ± 883.30	12,460.00 ^ab^ ± 1327.53	14,055.00 ^c^ ± 1911.033	0.048
snoRNA	549.67 ^a^ ± 32.50	624.33 ^a^ ± 18.56	775.67 ^b^ ± 111.07	0.017
scaRNA	45.00 ± 10.15	50.33 ± 8.51	47.33 ± 10.79	0.808
lncRNA	245,212.67 ^b^ ± 21,287.17	200,859.00 ^a^ ± 14,486.99	166,449.00 ^a^ ± 17,072.84	0.005
snRNA	193.33 ± 12.09	224.00 ± 46.57	254.67 ± 22.75	0.126
miRNA	722.67 ± 89.20	625.33 ± 35.16	622.33 ± 55.47	0.172
Mt_tRNA	1341.00 ± 154.70	1573.33 ± 197.06	1442.00 ± 228.50	0.403

^a,b,c^ mean within the same row with different superscripts differ (*p* < 0.05).

## Data Availability

The original contributions presented in this study are included in the article; further inquiries can be directed to the corresponding authors.
